# Comparison of antibacterial activity and biocompatibility of non-leaching nitrofuran bone cement loaded with vancomycin, gentamicin, and tigecycline

**DOI:** 10.1186/s13018-023-04055-2

**Published:** 2023-08-04

**Authors:** Zhe Gao, Yang Xu, Yuchen Kan, Hailong Li, Rui Guo, Luyang Han, Wenhan Bu, Jianjun Chu

**Affiliations:** 1 Department of Orthopedics, The Second People’s Hospital of Hefei, Hefei Hospital Affiliated to Anhui Medical University, Hefei, 230011 Anhui China; 2 School of Food and Biological Engineering, Hefei University of Technology, Hefei, 230009 Anhui China; 3 Department of Orthopedics, The Second People’s Hospital of Fuyang, Fuyang, 236000 Anhui China

**Keywords:** Non-leaching antibacterial bone cement, Antibiotic-loaded bone cement, Vancomycin, Gentamicin, Tigecycline

## Abstract

**Background:**

Non-leaching antibacterial bone cement can generate long-term antibacterial activity, it cannot treat serious infections that have occurred like antibiotic-loaded bone cement. Currently, the antibacterial activity and biocompatibility of non-leaching cement when loaded with antibiotics have yet to be determined.

**Methods:**

Non-leaching antibacterial nitrofuran bone cement (NFBC) specimens were prepared with low-dose and high-dose antibiotics. The antibacterial activity and biocompatibility of NFBC loaded with vancomycin, gentamicin, and tigecycline were compared. The agar diffusion method was employed to observe the inhibition zone of the samples against two bacterial strains from day one to day seven. The CCK-8 assay and acute liver and kidney toxicity test were conducted to assess the effects of the samples on mouse embryo osteoblast precursor cells and C57 mice, respectively.

**Results:**

Gentamicin-loaded cement exhibited the most potent antibacterial activity, effectively inhibiting both bacterial strains at a low dose. Tigecycline-loaded cement demonstrated superior biocompatibility, showing no acute liver and kidney toxicity in mice and minimal cytotoxicity to osteoblasts.

**Conclusions:**

NFBC loaded with gentamicin, vancomycin, and tigecycline not only maintains sustained antibacterial activity but also exhibits excellent biocompatibility.

**Supplementary Information:**

The online version contains supplementary material available at 10.1186/s13018-023-04055-2.

## Background

Antibiotic-loaded bone cement (ALBC) has been widely used in clinical practice, it has also presented several issues, such as the burst release of antibiotics, which typically fall below the minimum inhibitory concentration after one week of release and can easily lead to bacterial resistance [1–6]. In addition to physically mixing antibiotics into bone cement, antibacterial groups can also be covalently bonded to the bone cement matrix and permanently modify the surface properties of the cement, which is known as non-leaching antibacterial bone cement [7, 8]. Unlike ALBC, fixed antibacterial agents are usually connected to the polymer skeleton via ester or amide bonds, which exhibit strong chemical stability and can produce long-term antibacterial effects [9, 10].

Quaternary ammonium salts are the most commonly used fixed antibacterial groups [11, 12]. Recent studies have shown that heterocyclic compounds, such as nitrofuran and benzothiazole, exhibit good antibacterial activity [13–16]. When these heterocyclic compounds are covalently incorporated into bone cement to prepare benzothiazole bone cement or nitrofuran bone cement (NFBC), they both exhibit excellent antibacterial activity. Although non-leaching antibacterial bone cement exhibits long-term antibacterial ability, it is difficult to address acute infections that have already occurred.

In this study, our objective is to incorporate two commonly used antibiotics, vancomycin, and gentamicin, as well as a new broad-spectrum antibiotic, tigecycline, into the non-leaching NFBC and investigate the antibacterial activity, cytotoxicity, and in vivo biocompatibility of these composite cements. To the best of our knowledge, the properties of non-leaching antibacterial bone cement upon antibiotics, including antimicrobial activity and biocompatibility, have not been previously evaluated. Therefore, we asked the following questions: (1) Do gentamicin, vancomycin, and tigecycline loaded in non-leaching NFBC exhibit antibacterial activity? (2) What is the duration of antimicrobial activity and biocompatibility of these composite cements?

## Methods

### Materials

The NFBC used in this study was p(10% NFMA-co-MMA). All bone cement samples were composed of NFBC powder and methyl methacrylate (MMA) liquid monomer in a 2:1 ratio. The NFBC was prepared using literature-reported methods [16]. Briefly, p(10% NFMA-co-MMA) is obtained by polymerization of 10% 5-nitrofurfuryl alcohol methacrylate (NFMA) with MMA. *Staphylococcus aureus* (*S. aureus*, ATCC 25923) and *Escherichia coli* (*E. coli*, ATCC 25922) were obtained from The Second People's Hospital of Hefei, China. C57 mice were purchased from the Experimental Animal Center of Anhui Medical University, Hefei, China. Experimental procedures were approved by biomedical ethics committee.

Cylindrical cement samples with a diameter of 6 mm and a height of 12 mm were used to prepare extract for biocompatibility studies. Disk-shaped samples with a diameter of 6 mm and a height of 3 mm were employed for conducting antimicrobial experiments. For low-dose antibiotic-loaded cement, antibiotics were added at concentrations of 2.5 wt%, 5 wt%, respectively. For high-dose antibiotic-loaded cement, antibiotics were added at concentrations of 10 wt%. All these were grouped according to Table 1.

**Table 1:** Composition of antibiotic-loaded NFBC

	wt%	Weight of antibiotics per 40 g cement (g)
Low dose	2.5	1
	5	2
High dose	10	4

### Antibacterial activity (inhibition zone)

*Staphylococcus aureus* was used as the Gram-positive bacterial strain and *E. coli* as the Gram-negative bacterial strain to study the antibacterial activity of the bone cement. A total of 100 μL of bacterial solution with a 0.5 MacFarland turbidity standard was evenly spread on the agar plate. Disks of the test materials were prepared using a Teflon template (6 mm diameter 3 mm height). Three disks are placed in each medium, each disk must be more than 24 mm apart at the center and the disk must be more than 15 mm from the inside of the plate. The plates were incubated for 24 h at 37 °C. Subsequently, the antibacterial activity was evaluated by measuring the inhibition zone formed around the bone cement sample. Five agar plates were prepared for each experiment.

### Cytotoxicity (CCK-8 assay using mouse embryo osteoblast precursor cells (MC3T3-E1))

Approximately 5 × 10^3^ cells (MC3T3-E1) were seeded per well in a 96-well plate and cultured in MEM supplemented with 10% FBS as described above. The original culture medium was then removed and one hundred microliters of the different dilutions of the test material extract were co-cultured with MC3T3-E1, in three replicates. Cells cultured with 10% serum alone (i.e., cells unexposed to test material) were considered as the cell control. The morphological changes, OD values, and relative growth rate (RGR) of the osteoblasts were observed and recorded on days 1, 3, and 5 to evaluate the in vitro biocompatibility of the cement. The absorbance at 450 nm of every well was measured by a microplate reader. RGR was calculated according to equation:

$${\text{RGR}} = \frac{{{\text{OD}}_{T} - {\text{OD}}_{R} }}{{{\text{OD}}_{N} - {\text{OD}}_{R} }} \times 100$$

In the formula, OD_*T*_ is the absorbance of the experimental group, OD_*N*_ is the absorbance of the negative control group, and OD_*R*_ is the absorbance of the cell-free medium.

### Histopathological examination

Forty healthy SPF C57 mice of about 20 g were divided into 4 groups. Normal saline extracts of the three high-dose antibiotic bone cements were injected into the abdominal cavity at a dose of 50 ml/kg, respectively, and saline was used as a negative control group. General state (temperature, respiration, appetite, exercise, etc.) and toxic manifestations (vomiting, diarrhea, convulsions, etc.) of the animals were observed before administration and on day 1, 2, and 3 after administration. On the 3rd day (72 h later), the animals were sacrificed and the liver and kidney were taken out for pathological observation.

### Statistical analysis

The data of inhibition zone, cell absorbance, and relative proliferation rate were analyzed using SPSS 25.0 software. The quantitative data were expressed as mean ± standard deviation (*x* ± *s*). Statistical analysis was performed using one-way analysis of variance, and pairwise comparison of means was conducted using t-tests. Differences were considered significant when *p* < 0.05.

## Results

### Antibacterial activity

**Fig. 1:**
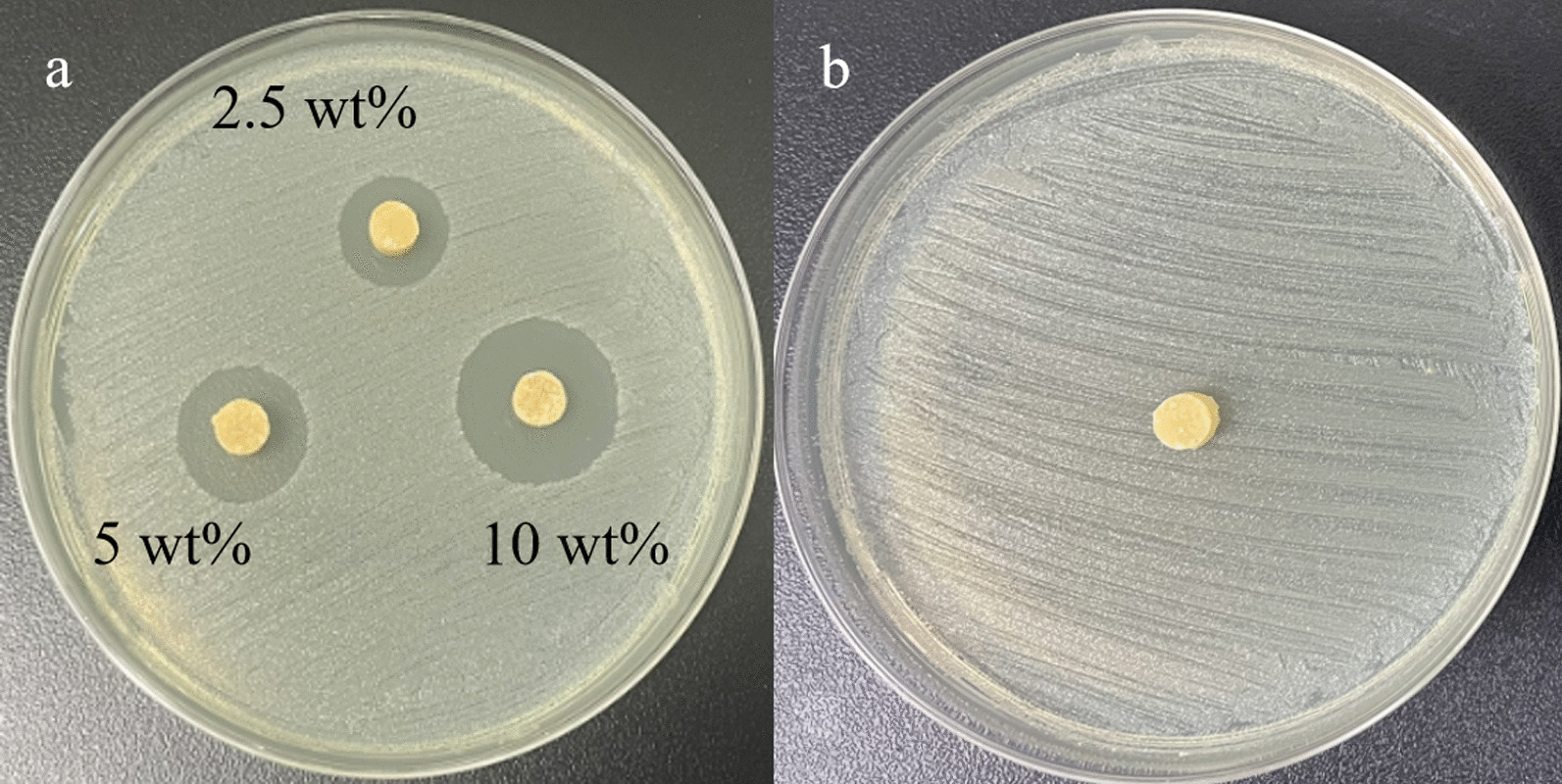
The inhibition zone produced by the sample against *Staphylococcus aureus* during the first day. **a** NFBC loaded with 2.5 wt%, 5 wt%, and 10 wt% vancomycin, respectively. **b** NFBC without loading any antibiotics

The agar diffusion method was used in the experiment for 7 consecutive days. The results showed that no inhibition zone was produced in the blank group (Fig. 1).

**Fig. 2:**
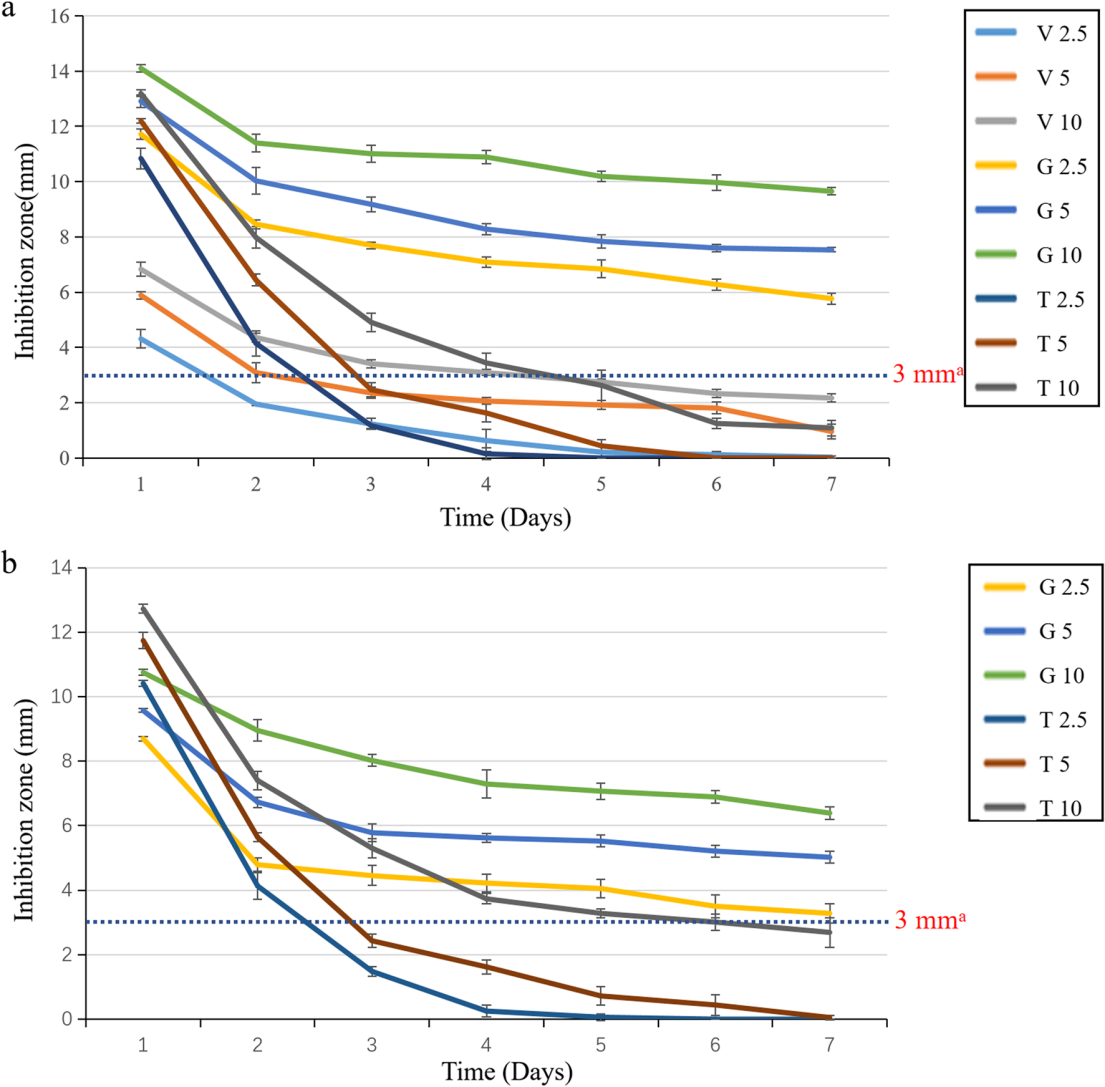
The inhibition zone of antibiotic-loaded NFBC with time. **a** Different antibiotic-loaded cement against *S. aureus*. **b** Different antibiotic-loaded cement against *Escherichia coli*. ^a^The inhibition zone less than 3 mm indicates lack of antibacterial activity

As shown in Fig. 2, the group loaded with vancomycin, gentamicin, and tigecycline all produced inhibition zone larger than 3 mm on the first day, and gentamicin-loaded cement had the largest inhibition zone against *S. aureus*, the vancomycin-loaded cement had the smallest zone against *S. aureus* (*p* < 0.05); while tigecycline-loaded cement had the largest inhibition zone against *E. coli* (*p* < 0.05). The inhibition zone becomes larger with the increase in antibiotics (*p* < 0.05), which proves that all groups are sensitive to *S. aureus* and *E. coli*, and the diameter of inhibition zone is positively correlated with the amount of antibiotics added.

The inhibition zone in each group was greatest on the first day and gradually decreased over 7 days (*p* < 0.05). When against with *S. aureus*, the effective bactericidal time (the inhibition zone bigger than 3 mm) of bone cement loaded with 2.5 wt%, 5 wt %, and 10 wt % vancomycin was 1 day, 2 days, and 4 days, respectively; the effective bactericidal time of bone cement loaded with different amounts of gentamicin was 7 days; the effective bactericidal time of 2.5 wt %, 5 wt %, 10 wt % tigecycline-loaded cement was 2 days, 2 days, and 4 days, respectively. When against with *E. coli*, the effective bactericidal time of cement loaded with different concentrations of gentamicin was 7 days; the bactericidal time of bone cement loaded with 2.5 wt %, 5 wt %, and 10 wt % tigecycline was 2 days, 2 days, and 6 days. Surprisingly, bone cement loaded with gentamicin not only has a longer antibacterial time, but also has a larger inhibition zone.

### Cytotoxicity (CCK-8 assay)

**Fig. 3:**
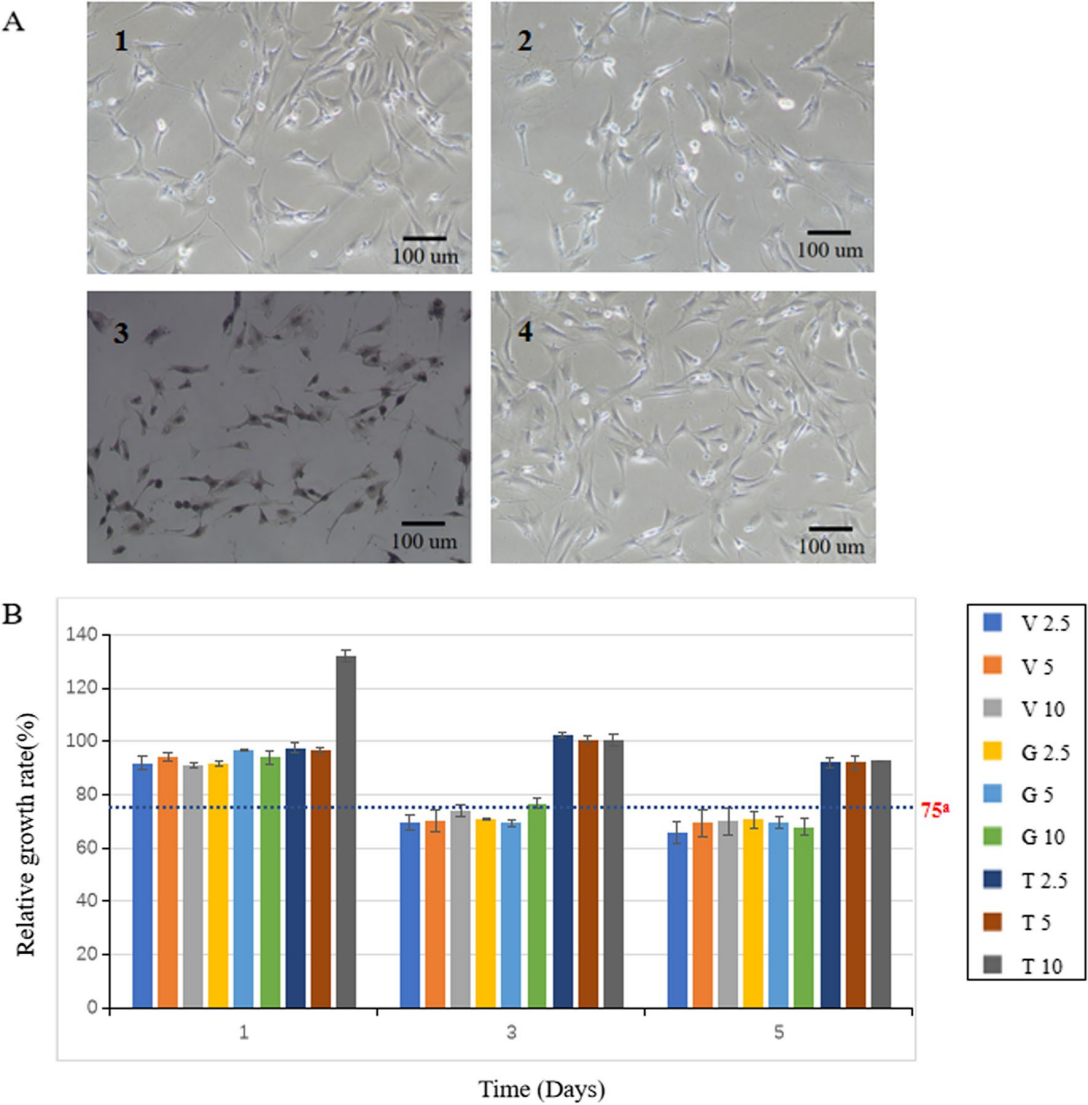
Cytotoxicity of NFBC loaded with three antibiotics at different doses. **a** Image of MC3T3-E1 cells incubated with different extracts (observed on the 5th day), 1: 10 wt% vancomycin group, 2: 10 wt% gentamicin group, 3: 10 wt% tigecycline group, 4: negative control group. **b** Relative growth rate of cells. ^a^Relative growth rate higher than 75% indicates no toxicity

The cells (MC3T3-E1) were mainly spindle-shaped and irregular triangles without necrotic cell debris when observed on the 1st day, 3rd day, and 5th day (Fig. 3a). The OD of each group continued to rise (*p* < 0.05), indicating that the cells were in a state of proliferation, and the relative growth rate (RGR) is calculated by OD value (Fig. 3b). Then, according to the cytotoxicity grading standard, the three antibiotic groups had varying degrees of inhibitory effects on osteoblast proliferation with the prolongation of the experimental period (*p* < 0.05), the vancomycin group and the gentamycin group showed no toxicity on the first day, slight toxicity began to appear on the third day, and both showed slight toxicity on the fifth day. The tigecycline group had the lowest degree of inhibition on osteoblasts, and no toxic reaction was observed in the experiment.

### Histopathological examination

**Fig. 4:**
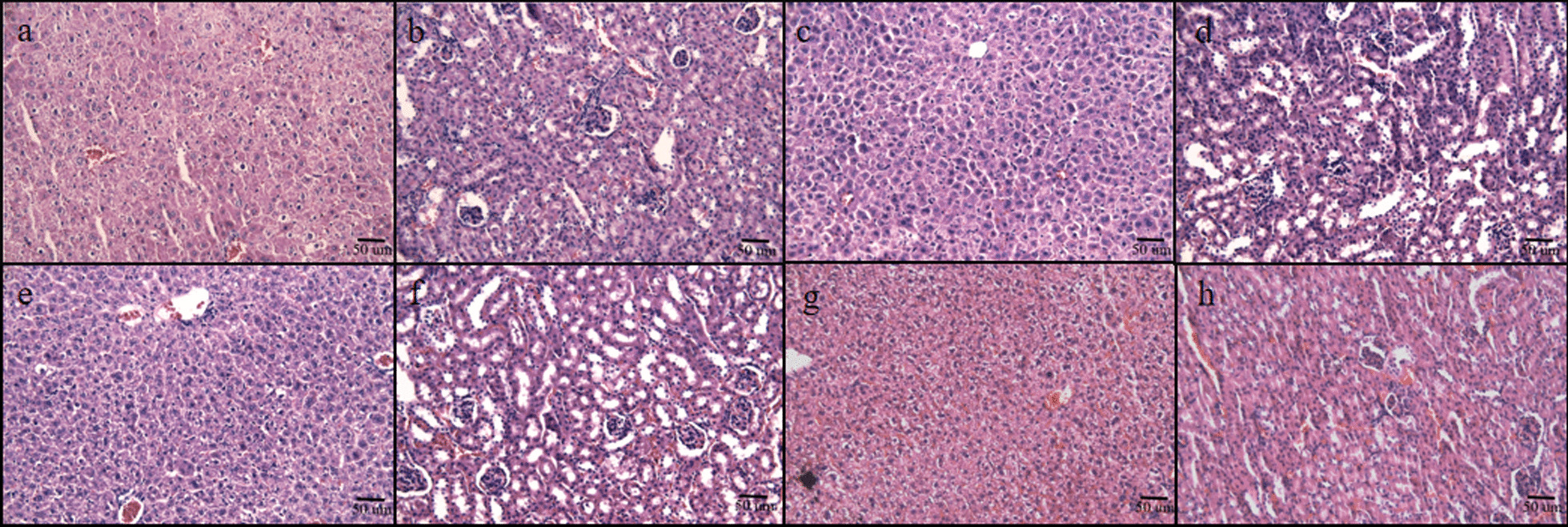
Liver and kidney sections of C57 mice injected with high-dose antibiotic-loaded NFBC extract. **a** Liver, 10 wt% vancomycin-loaded cement extract, **b** kidney, 10 wt% vancomycin-loaded cement extract, **c** liver, 10 wt% gentamicin-loaded cement extract, **d** kidney, 10 wt% gentamicin-loaded cement extract, **e** liver, 10 wt% tigecycline-loaded cement extract, **f** kidney, 10 wt% tigecycline-loaded cement extract, **g** liver, negative control group, **h** kidney, negative control group

The mice were observed three days after intraperitoneal injection of the extract and all groups of mice were in good condition with no signs of toxicity and no mice died. No abdominal adhesions were observed when the mice were executed and the pathological sections showed no obvious degeneration or necrosis of the liver and kidney cells and tissues of the mice in each group, with no toxic changes (Fig. 4). This indicates that the gentamicin, vancomycin, and tigecycline groups had no acute hepatorenal toxicity in c57 mice.

## Discussion

Burst release leads to a short antibacterial time for ALBC. Recently, various non-leaching antibacterial bone cements have been developed to achieve more durable antibacterial properties. For instance, Xie et al. introduced an antibacterial bone cement containing non-leaching furanone derivatives. Leaching experiments demonstrated that this bone cement did not release antibacterial components, yet it exhibited effective antibacterial properties against common clinical bacteria [14]. Similarly, Fu et al. [16] developed a non-leaching antibacterial bone cement using nitrofuran, which displayed excellent antibacterial activity in direct contact experiments. In this study, we observed that NFBC without antibiotic addition showed no inhibition zone (Fig. 1b), aligning with Xie’s findings. This indicates that NFBC does not release antibacterial components. Therefore, ALBC primarily exerts a potent antibacterial effect against acute infections, while the non-leaching antibacterial NFBC incorporates antibacterial groups covalently into the polymer, enabling long-term antibacterial properties. Although NFBC demonstrates prolonged antibacterial abilities, it faces challenges in addressing pre-existing acute infections. Thus, we aim to incorporate antibiotics into NFBC to investigate its antibacterial release properties against acute infections.

The overuse of gentamicin and vancomycin has led to an increased risk of the emergence of resistant strains [17, 18]. Therefore, the exploration and utilization of bone cements with novel antibiotic additions have been pursued as a strategy to address bacterial resistance [19]. Tigecycline, a new semi-synthetic glycylcycline antibiotic, is a derivative of the tetracyclines. It is specifically designed to treat serious infections caused by numerous existing resistant strains, including vancomycin-resistant enterococci, methicillin-resistant *S. aureus*, and various multidrug-resistant gram-negative bacteria [20, 21]. However, there are fewer reports of tigecycline-loaded cements. Therefore, in this study, two classical antibiotics, gentamicin, and vancomycin, were selected for study, and tigecycline-loaded cements were also investigated.

**Fig. 5:**
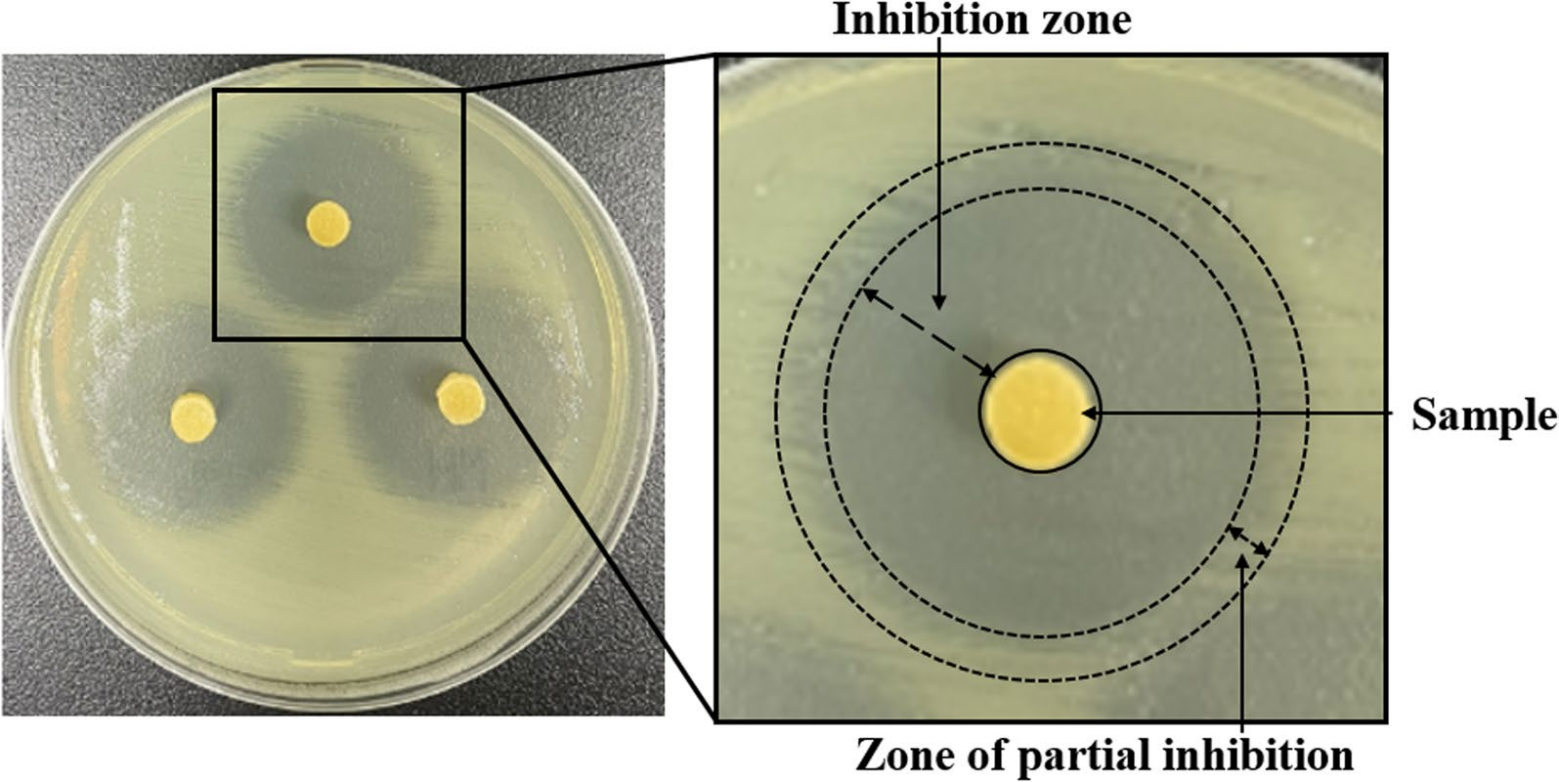
Schematic representation of inhibition zone evaluation test. Image showing the inhibition zone of gentamicin-loaded NFBC against *E. coli*. The magnification inset underlines the halo characteristics and in particular the partial inhibition zone

In this study, the definition of the inhibition zone is the distance between the test disk and bacterial growth, as illustrated in Fig. 5. Previous studies have identified translucent areas extending from the edge of the zone, known as the zone of partial inhibition [22, 23]. This phenomenon is more prominent in the inhibition zone produced by gentamicin against *E. coli* or tigecycline against *S. aureus*. It occurs when the antibiotic exerts partial inhibition on the bacteria. The zone of partial inhibition is not included in the measurement of the zone of inhibition. It is widely accepted that a bacterial strain is considered susceptible to an antibiotic if it produces a zone larger than 3 mm, while it is regarded as resistant if it does not generate a halo [22, 23]. The size of the zone is influenced by various factors, primarily related to the antibacterial properties of the drug itself and the diffusion rate of the drug on the agar [24, 25].

Although many studies currently assess the antibacterial performance of bone cement specimens by measuring the inhibition zone after approximately 24 h [2, 26, 27], we believe it is valuable to evaluate the antibacterial effect of these materials over an extended period. This approach allows for an exploration of whether the antibacterial properties continue to be effective during the susceptible period after surgery, rather than providing only a short-term, 24 h evaluation.

The results indicated that the blank group did not exhibit any inhibition zone, while all antibiotic-loaded bone cements demonstrated the ability to produce an inhibition zone. The size of the zone was positively correlated with the amount of antibiotic added, with the high-dose group exhibiting the strongest effect. However, the size of the inhibition zone decreased as the experimental period was prolonged.

The burst release behavior was most obvious in tigecycline-loaded cement. Initially, the inhibition zone of tigecycline was similar to that of gentamicin on the first day, but it rapidly decreased on the second day. By the third day, the two low-dose tigecycline-loaded cements (1 g and 2 g tigecycline per 40 g cement) lost their sensitivity to *S. aureus* and *E. coli*. In contrast, the release of gentamicin from the NFBC was more sustained, and all groups remained sensitive to both bacteria throughout the experimental period (Fig. 2).

The antibacterial activity of vancomycin-loaded cement was notably weaker than that of the other two cements, resulting in a smaller inhibition zone. This could be attributed to the characteristics of vancomycin as a glycopeptide antibiotic, which exhibits poor diffusion and produces only a small zone even at high doses [28]. The difference in elution capabilities may be attributed to the significantly higher molecular weight of vancomycin compared to gentamicin and tigecycline [23, 24, 29]. These findings suggest that the choice of antibiotic and its dosage in bone cements can significantly impact the size and duration of the inhibition zone, with implications for their long-term effectiveness in preventing bacterial infections.

When an orthopedic implant is applied in clinical settings, the primary consideration is biocompatibility [30]. It should be non-toxic, without carcinogenic or teratogenic risks, and should not cause immune rejection. Currently, negative effects of gentamicin and vancomycin on osteoblasts have been reported [31, 32], while studies have shown that tigecycline is safe and effective in animal experiments and clinical studies, particularly in the treatment of skin, soft tissue, intestinal, and abdominal conditions [33].

The CCK-8 assay demonstrated proliferative cell growth in all groups (Fig. 3a). However, based on the relative growth rate and subsequent cytotoxicity grading analysis, the gentamicin and vancomycin groups exhibited varying degrees of inhibition on osteoblasts, which increased from non-toxic to mildly toxic by day 5 (Fig. 3b). This is consistent with reports, indicating that both vancomycin and gentamicin can adversely affect the viability of osteoblasts. We were surprised to find that tigecycline-loaded cement showed no toxicity. The c57 mice in each group were in good condition before and after injection of the extract, and no obvious toxic reactions occurred. The morphology of liver and kidney cells and tissues in each group was normal, without apparent degeneration or necrosis, indicating no significant acute toxicity to the experimental animals in any of the groups. Taken together, the tigecycline-loaded cement exhibited the best biocompatibility.

## Conclusions

In this study, we observed that non-leaching NFBC loaded with antibiotics exhibited strong antibacterial activity and good biocompatibility. Gentamicin proved to be an effective option for treating infections, showing persistent antibacterial activity even at low doses. Furthermore, tigecycline-loaded NFBC demonstrated satisfactory biocompatibility. These findings emphasize the potential of antibiotic-loaded NFBC as a viable solution for addressing infections in orthopedic implants while ensuring patient safety.

### Supplementary Information

**Additional file 1. Figure S1:** Image of inhibition zone against S. aureus for 7 days. (**a**) NFBC loaded with vancomycin. (**b**) NFBC loaded with gentamicin. (c) NFBC loaded with tigecycline.

**Additional file 2. Figure S2:** Image of inhibition zone against E. coli for 7 days. (**a**) NFBC loaded with gentamicin. (**b**) NFBC loaded with tigecycline.

## Data Availability

All data generated or analyzed during this study are included in this published article and its Additional file 1 and 2.
