# Supplementary material for: Comparison of antibacterial activity and biocompatibility of non-leaching nitrofuran bone cement loaded with vancomycin, gentamicin, and tigecycline
Source: J Orthop Surg Res. 2023 Aug 4;18:569. doi: 10.1186/s13018-023-04055-2 (PMC10403827; doi:10.1186/s13018-023-04055-2)
Supplement: Supplementary file 1 — Additional file 1. Figure S1: Image of inhibition zone against S. aureus for 7 days. (a) NFBC loaded with vancomycin. (b) NFBC loaded with gentamicin. (c) NFBC loaded with tigecycline. [file 13018_2023_4055_MOESM1_ESM.pdf]

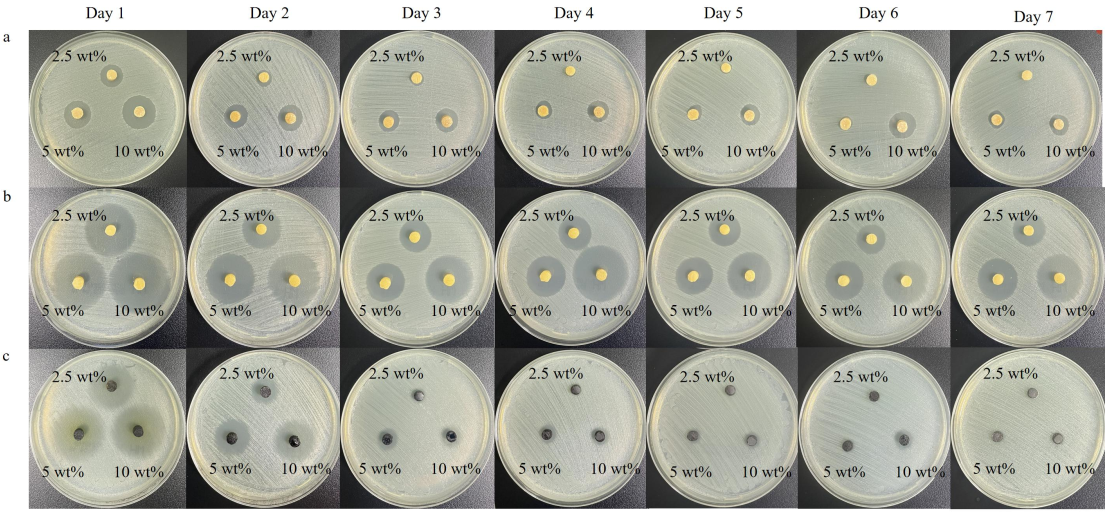

Additional file 1: Figure S1: Image of inhibition zone against *S. aureus* for 7 days. (a) NFBC loaded with vancomycin. **(b)** NFBC loaded with gentamicin. **(c)** NFBC loaded with tigecycline.
